# Lignin Particle Size Affects the Properties of PLA Composites Prepared by In Situ Ring-Opening Polymerization

**DOI:** 10.3390/polym16243542

**Published:** 2024-12-19

**Authors:** Sofia P. Makri, Eleftheria Xanthopoulou, Panagiotis A. Klonos, Alexios Grigoropoulos, Apostolos Kyritsis, Ioanna Deligkiozi, Alexandros Zoikis-Karathanasis, Nikolaos Nikolaidis, Dimitrios Bikiaris, Zoi Terzopoulou

**Affiliations:** 1 Creative Nano PC, 43 Tatoiou, Metamorfosi, 14451 Athens, Greece; s.makri@creativenano.gr (S.P.M.); a.grigoropoulos@creativenano.gr (A.G.); i.deligkiozi@creativenano.gr (I.D.); a.karathanasis@creativenano.gr (A.Z.-K.); 2 Laboratory of Polymer and Colors Chemistry and Technology, Department of Chemistry, Aristotle University of Thessaloniki, 54124 Thessaloniki, Greece; exanthoa@chem.auth.gr (E.X.); pklonos@central.ntua.gr (P.A.K.); nfnikola@chem.auth.gr (N.N.); 3 Dielectrics Research Group, Department of Physics, National Technical University of Athens, Zografou Campus, 15780 Athens, Greece; akyrits@central.ntua.gr

**Keywords:** organosolv, lignin, poly(lactic acid), ultrasonication, composites

## Abstract

The present work focuses on the synthesis and characterization of biobased lignin-poly(lactic) acid (PLA) composites. Organosolv lignin, extracted from beechwood, was used as a filler at 0.5, 1.0, and 2.5 wt% loadings, with ultrasonication reducing the lignin particle size to ~700 nm. The PLA–lignin composites were prepared via in situ ring-opening polymerization (ROP) of L-lactide in the presence of lignin. This method ensured uniform lignin dispersion in the PLA matrix due to grafting of PLA chains onto lignin particles, preventing aggregation. Strong polymer–filler interactions were confirmed through spectroscopic analysis (FTIR and XPS) and their effects on static and dynamic glass transitions (DSC). These interactions enhanced mechanical properties, including a two-fold increase in tensile strength and elongation at 1 wt% lignin. Crystallization was suppressed due to shorter PLA chains, and a 15% drop in dynamical fragility was observed via Broadband Dielectric Spectroscopy (BDS). Antioxidant activity improved significantly, with PLA–2.5% ultrasonicated organosolv lignin reducing DPPH• content to 7% after 8 h, while UV-blocking capability increased with lignin content.

## 1. Introduction

Amid the increasing demand for sustainable materials, polymer science has embraced innovative and eco-friendly practices to advance the development of biodegradable alternatives to traditional petroleum-based plastics like polyethylene, polypropylene, and poly(ethylene terephthalate). Polymers like poly(lactic acid) (PLA) that originate from renewable resources are considered excellent candidates for the replacement of fossil-based plastics [1]. PLA is a versatile compostable polymer derived from corn starch, sugarcane, or sugar beet [2]. This aliphatic polyester exhibits remarkable properties such as biocompatibility and melt processability and can be used in various applications, ranging from medical devices to packaging materials [3]. However, PLA does face certain limitations including modest mechanical strength, sensitivity to moisture, limited antioxidant capability, low thermal resistance, and very slow crystallization [4]. These constraints have led to a drive for innovative strategies aimed at enhancing PLA’s mechanical, thermal, and barrier properties by incorporating lignocellulosic biomass-derived fillers, such as cellulose and lignin [5,6]. PLA is a semicrystalline polymer; thus, the performance improvement via filler reinforcement may be both direct and indirect, e.g., via the increase in the degree of crystallinity [7,8,9,10].

Lignin is a ubiquitous natural polymer, ranking as the second most abundant component of biomass. It has a three-dimensional polyphenolic, hydrophobic backbone that is formed via the cross-linking of three primary p-(hydroxypropenyl) phenols, commonly referred to as lignols, namely, coniferyl (G-units), sinapyl (S-units), and p-coumaryl (H-units) alcohol. Moreover, lignin possesses a range of surface functional groups such as hydroxyl, carbonyl, and carboxyl groups that further contribute to its key characteristics. Therefore, lignin is recognized as an excellent biobased reinforcing filler that can also enhance antioxidant, antimicrobial, flame retardant, and barrier capability [11,12,13].

Lignin, a byproduct of pulp and paper production, is typically separated from cellulose using methods like the kraft and sulfite processes. Organosolv extraction, a more eco-friendly alternative, has gained attention for yielding higher-quality lignin [14]. Extracted under hydrothermal conditions with water/alcohol mixtures, sometimes with acid catalysts, organosolv lignin features a less modified structure, lower molecular weight, more hydroxyl groups, and greater uniformity, making it ideal for polymer applications [15,16,17,18]. In our previous work, organosolv lignin extracted from beechwood biomass was used as a filler in PLA-based composites [19]. First, organosolv lignin was ball milled to decrease the particle size. Afterward, the ground lignin was dispersed in PLA through solvent casting to prepare PLA–lignin masterbatches with different lignin content. Finally, PLA–lignin composites with loadings of 0.5, 1.0, and 2.5 wt% were produced by melt mixing of the masterbatches with PLA. All composites showed an increasing trend in antioxidant activity with higher lignin loadings superior to neat PLA, increasing with lignin content. On the other hand, a ball milling pretreatment step of organosolv lignin had minimal impact on the size of lignin, which remained between 1 and 2 μm, nonetheless a slightly improvement was observed in the the composites’ properties.

Melt mixing is the most conventional method for preparing PLA–lignin composites, having provided valuable insights into their potential applications [6]. However, it is often difficult to achieve a uniform dispersion of lignin in the PLA matrix via melt mixing, primarily because lignin tends to form aggregates. The poor lignin dispersion leads to weak interfacial interactions with PLA and as a result to reduced mechanical integrity of the respective composites [20,21,22,23,24]. Several studies have shown that decreasing the size of lignin particles to the nanoscale can enhance its compatibility with PLA [22,25,26,27,28,29,30,31,32] and produce PLA–lignin nanocomposites with improved mechanical and physicochemical properties [31,33,34]. Ring-opening polymerization (ROP) is a highly efficient and widely used method for synthesizing biodegradable and renewable polymers from cyclic monomers, such as lactides, caprolactones, and cyclic carbonates. ROP offers precise control over polymer molecular weight and architecture, making it a preferred method for producing PLA with tailored properties [35]. Recent advancements in ROP have explored novel catalysts, including metal-free organocatalysts and biocatalysts, which offer reduced environmental impact and higher efficiency under mild conditions [36]. Additionally, ROP allows for the grafting of PLA chains directly onto lignin particles via their hydroxyl functional groups, resulting in covalent bonding and improved dispersion. Unlike conventional melt mixing, in situ ROP facilitates uniform filler integration while maintaining the structural integrity of both the polymer and filler [25]. This innovative approach has been further enhanced by employing nanoscale fillers, such as ultrasonicated lignin, which increase the availability of active sites and improve interfacial interactions. These developments underscore the versatility of ROP as a tool for synthesizing high-performance, sustainable polymer composites.

We have also reported the first example of synthesizing PLA–kraft lignin composites via in situ ROP reactive extrusion, a method that could be upscaled for industrial applications [25]. Notably, the composites with a 0.5 wt% content synthesized via in situ ROP exhibited more uniform and finer lignin dispersion, improved crystallization, mechanical, antioxidant, and UV barrier properties compared with the ones produced via melt mixing. This was attributed to the formation of covalent bonds (grafting) between PLA and lignin during the in situ ROP reactive process. The above properties were further enhanced when kraft lignin nanoparticles were instead used. This further promoted PLA–lignin grafting by virtue of the lignin nanoparticles’ higher surface area and finer dispersion [27,37,38,39,40,41].

In this study, we present the synthesis of PLA-based composites with organosolv lignin via in situ ROP to exploit its ability to improve dispersion and the respective composites’ final properties. Organosolv lignin, either as received after extraction from beechwood biomass (oL) [16] or after ultrasonication (US) treatment ((US)oL), was used as a filler at three different concentrations of 0.5, 1, and 2.5 wt%. Our work highlights the significance of lignin particle size, which can be reduced by ultrasonication. A comparative analysis elucidates how the particle size influences the processability and the properties of the PLA–lignin composites. The physicochemical and morphological properties of the prepared composites were investigated via Fourier transform infrared spectroscopy (FTIR), Differential Scanning Calorimetry (DSC), Broadband Dielectric Spectroscopy (BDS), Polarized Electron Microscopy (PLM), Scanning Electron Microscopy (SEM), and Transmission Electron Microscopy (TEM), Nuclear Magnetic Resonance (NMR), and X-ray Photoelectron (XPS) spectroscopy. Finally, the mechanical, antioxidant, and optical properties of the composites were also evaluated.

## 2. Materials and Methods

### 2.1. Materials

As-received oL with a hydrodynamic diameter of approximately 1 μm, as measured by Dynamic Light Scattering (DLS) in water (Figure S1), M_n_ = 640 g/mol, Ð = 3.67, 64.3% syringyl, and 34.5% guaiacyl units was prepared at the Laboratory of Chemical and Environmental Technology, Department of Chemistry (Aristotle University of Thessaloniki, AUTH), as previously described by Margellou et al. [16]. The US-treated (US)oL with a hydrodynamic diameter of approximately 700 nm (Figure S1) was produced and provided by Creative Nano PC (Athens, Greece) through a process described in European Patent No. EP4471093 [42]. L-lactide (purity > 99.5%, melting range 96–100 °C) with the trade name PURASORB^®^ L was supplied by Corbion N.V. (Amsterdam, The Netherlands). All chemicals including 2,2-diphenyl-1-picrylhydrazyl (DPPH radical, 95%) that was used for the antioxidant activity tests, were purchased from Sigma-Aldrich (St. Louis, MO, USA) and used with no further purification.

### 2.2. Synthesis of PLA–oL and PL–(US)oL Composites via ROP

The PLA–oL/(US)oL composites were synthesized via ROP of L-Lactide. Prior. Firstly, PLA was dried under vacuum for 24 h and then placed in a round bottom flask containing the appropriate amount of oL or (US)oL, 0.1% mol of 1-dodecanol dispersion in acetone (only for the neat PLA synthesis), and Sn(Oct)_2_ catalyst (400 ppm based on L-Lactide). The apparatus was evacuated and refilled with N_2_ three times to remove oxygen. The reacting mixture was heated for 1 h at 170 °C and 30 min at 190 °C, under N_2_ flow (50 mL/min). To further increase the molecular weight of the produced PLA–oL/(US)oL composites and remove undesired products (i.e., oligomers and unreacted monomer), a high vacuum (∼5.0 Pa) was slowly applied over a period of 15 min. The final samples were collected from the reactor and washed with methanol. Films of each sample were prepared by compression molding using an Otto Weber Type PW 30 hydraulic press (Stuttgart, Germany) connected with an Omron E5AX Temperature Controller (Kyoto, Japan), at a temperature of 190 ± 5 °C and pressure of 50 bar. After compression molding, the films were rapidly cooled to room temperature. All the films had a similar thickness of approximately 0.25 mm.

### 2.3. Characterization Methods

#### 2.3.1. Particle Size Distribution of Lignin

The average particle size of the as received organosolv lignin and the ultrasonicated organosolv lignin was measured by DLS on a Litesizer 500 instrument (Anton Paar, Graz, Austria). Prior to the DLS measurements, the lignin powders were dispersed in water via ultrasonication for 5 min at a concentration of 100 ppm. This measurement highlights the impact of ultrasonication on the particle size distribution, a critical parameter influencing the dispersion quality and interaction potential of lignin within the PLA matrix.

#### 2.3.2. Intrinsic Viscosity Value Measurements

The intrinsic viscosity of the synthesized composites was measured with an Ubbelohde viscometer (Schott Gerate GMBH, Hofheim, Germany) at 25 °C, using chloroform as the solvent. The samples were heated at 50 °C for 20 min until complete dissolution. After cooling to room temperature, the solutions were filtered through a disposable Teflon filter (pore size 0.45 μm) to remove possible solid residues. The calculation of the intrinsic viscosity value of the polymer was performed applying the Solomon–Cuita Equation (1) of a single point measurement:

(1) $$\left[\eta \right]=\frac{{\left[2\left\{\frac{t}{t_{0}}-ln\left(\frac{t}{t_{0}}\right)-1\right\}\right]}^{\frac{1}{2}}}{c}$$

#### 2.3.3. Gel Permeation Chromatography (GPC)

The molecular weight was evaluated with an Agilent Technologies 1260 Infinity II LC Gel Permeation Chromatography (GPC) system consisting of a pump, a PL gel MIXED Guard column and two PLgel 5 μm MIXED-C columns, and an Agilent RID detector using an isocratic gradient. For the calibration, three poly(methyl methacrylate) (PΜΜA) standards of molecular weights between 600 and 1,000,000 g/mol were employed. The prepared solutions had a concentration of 1 mg/mL in chloroform (Sigma-Aldrich, St. Louis, MO, USA), and the injection volume was 20 μL with a flow rate of 1 mL/min at a temperature of 40 °C.

#### 2.3.4. Fourier-Transformed Infra-Red Spectroscopy (FTIR)

ATR-FTIR spectra of the prepared films were recorded on a Tension 27 FTIR spectrometer (Bruker, Billerica, MA, USA) equipped with a diamond ATR accessory with a spectral resolution of 4 cm^−1^ in the 4000–600 cm^−1^ range. FTIR spectra were analyzed with the OPUS software (version 5.2, Bruker, Billerica, MA, USA).

#### 2.3.5. Nuclear Magnetic Resonance (NMR)

Nuclear Magnetic Resonance (^1^H and ^13^C NMR) spectra were recorded in CDCl_3_ for investigating the structure of the synthesized polymers using an Agilent 500 spectrometer (Agilent Technologies, Santa Clara, CA, USA) at room temperature. Spectra were calibrated using the residual solvent peaks.

#### 2.3.6. X-Ray Photoelectron Spectroscopy (XPS)

X-ray Photoelectron Spectroscopy (XPS) analysis was conducted using a Kratos Analytical AXIS Ultra^DLD^ system, featuring an aluminum monochromatic X-ray source (λ_Ka_ = 8.3401 Å), under ultra-high vacuum conditions (10^−9^ Torr). Wide-scan spectra (full range) were recorded with a 160 eV pass energy on the analyzer, applying 105 W on the X-ray gun. Conversely, high-resolution (HR) regions were recorded with a 20 eV pass energy on the analyzer during a three-sweep scan, with 150 W applied to the X-ray gun. For spectrum calibration and to address surface charging, the C 1s peak at 284.6 ± 0.2 eV assigned to the C–C bonds was used. Spectra fitting processing involved the use of Shirley (non-linear) baselines for background subtraction, and the experimental curves were best fitted using a combination of Gaussian (70%) and Lorentzian (30%) distributions. Relative sensitivity factors (RSFs) for each element, essential for quantitative calculations, were derived from the Vision 2.2.10 software database.

#### 2.3.7. Transmission Electron Microscopy (TEM)

An FEI Tecnai G2 20 microscope (FEI, Hillsboro, OR, USA) was used to acquire TEM images at a 200 kV accelerating voltage. PLA films were embedded in epoxy resin (Araldite CY212) to prepare the TEM sample. The samples were cut with an ultramicrotome (DiATOME 45° diamond knife, DiATOME Ltd., Nidau, Switzerland) at an 80 nm thickness following a 48 h curing period. The thin pieces floating on the knife’s water surface were placed on carbon-coated grids and allowed to air dry for the entire night.

#### 2.3.8. Differential Scanning Calorimetry (DSC)

The thermal transitions were assessed by DSC in the temperature range from –10 to 190 °C in nitrogen atmosphere of high purity (99.9995%) by means of a TA Q200 DSC apparatus (TA Instruments, New Castle, DE, USA), calibrated with sapphires for heat capacity and indium metal for temperature and enthalpy. Pieces of the prepared samples of approximately 8 mg in mass were closed in aluminium Tzero TA pans. Upon erasing of the samples’ thermal history by a first heating up to 190 °C, two cooling–heating scans were performed, one involving a fast cooling rate (90–100 K/min, in the region of expected crystallization) and another involving a conventional cooling rate (20 K/min), in order to produce amorphous and semicrystalline polymers, respectively. Subsequent heating scans were performed at the fixed heating rate of 10 K/min.

#### 2.3.9. Broadband Dielectric Spectroscopy (BDS)

Using a Novocontrol BDS setup—a Novocontrol Quatro liquid nitrogen cryosystem (Novocontrol GmbH, Montabaur, Germany) in conjunction with an Alpha frequency response analyzer—the segmental mobility (dynamics) of the samples was investigated using BDS [43] in a nitrogen atmosphere. Initial amorphous samples were melted and quickly cooled to form a sandwich-like capacitor with a diameter of 20 mm and an electrode distance of approximately 1.5 mm (sample thickness). When heating at steps of 5 and 10 K, the complex dielectric permittivity, ε* = ε′ − i·ε″, was measured isothermally as a function of frequency, f, in the range of 10^−1^ to 106 Hz and with a temperature range of 10 to 120 °C.

#### 2.3.10. Polarized Light Microscopy (PLM)

For the PLM observations a polarized light microscope (Nikon, Tokyo, Japan, Optiphot-2), which was equipped with a Linkam THMS 600 heating stage (Surrey, UK), a Linkam TP 91 control unit, and a Jenoptic Gryphax Arktur camera with Gryphax software v 2.2.0.1234, was used.

#### 2.3.11. Tensile Testing 

Tensile tests were conducted according to ASTM D882 on the amorphous samples using the Shimadzu EZ Test, model ΕΖ-LX (Shimadzu, Kyoto, Japan), with a 5 mm/min crosshead speed. A Wallace cutting press was used to cut dog-bone-shaped tensile test specimens, with the central portions measuring 5 × 0.3 mm in thickness and 22 mm in gauge length. The mean values of Young’s modulus, tensile strength at yield and breakpoint, and elongation at break were calculated by averaging the results of at least three measurements for each sample. 

#### 2.3.12. DPPH Assay

Using UV-Vis spectroscopy, the antioxidant activity of the composites was determined by adding them to a solution of DPPH radical (DPPH•) and evaluating the reduction in absorbance of the solution and certain time intervals. By tracking the drop in absorbance of a DPPH• solution following incubation with the test sample, this technique assesses a substance’s capacity to scavenge free radicals. The control was a 0.079 mM DPPH• solution in EtOH that was left at room temperature in the dark for 16 h. At room temperature, films measuring the same (1 cm × 1 cm) were submerged in 3 mL of the DPPH• solution in EtOH. Using UV-Vis spectroscopy, the absorption decay at 517 nm was measured hourly to assess the composites’ antioxidant capacity. The specimens were kept in the dark between measurements. The remaining DPPH• content in the solution was calculated using Equation (2):

(2) $$Residual\;DPPH•content\;(\%)=100-100(A_{0}-\frac{A_{1}}{A_{0}})$$

where A_0_ is the absorbance of the control sample, and A_1_ is the absorbance in the presence of the films.

#### 2.3.13. UV Transmittance 

To evaluate the optical properties of PLA-based composites, diffuse reflectance spectroscopy (DRS) was employed using an Agilent Carry 60 spectrophotometer (Agilent Technologies, Santa Clara, CA, USA) equipped with a Harrick Video Barrelino DRA fiber optic coupler (Pleasantville, NY, USA) in the wavelength range of 200 to 800 nm. Baseline correction was performed using a BaSO_4_ standard.

## 3. Results and Discussion

### 3.1. Synthesis of PLA Composites with oL and (US)oL

**Figure 1:**
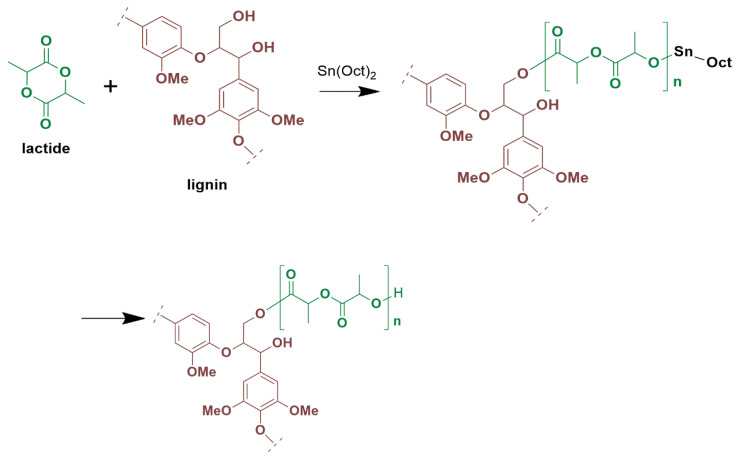
Possible reaction pathway of lactide polymerization in the presence of Sn(Oct)_2_, with lignin acting as a macroinitiator.

ROP is a method for synthesizing polymers from cyclic monomers were grafting of polymeric chains is achieved utilizing a hydroxyl source as an initiator [6]. In the ROP process, the initial step involves the activation of the Sn(Oct)_2_ catalyst through the hydroxyl source (ROH), resulting in the formation of a Sn–OR complex. Subsequently, Sn–OR complex interacts with the monomer, initiating the polymer chain formation via a coordination-insertion mechanism. During this stage, the polymer’s chain length increases as the monomers are consumed [39,44]. Lignins act as macroinitiators in the ROP of lactide by virtue of their abundant surface hydroxyl groups, leading to the grafting of PLA chains onto lignin particles [35,43,45,46], as shown in Figure 1. Therefore, it is expected that the synthesis reported herein using 0.5–2.5 wt% lignin loading should result in the covalent bonding between PLA and a significant number of mainly aliphatic lignin -OH groups, provided they are not sterically hindered. The most probable mechanism is presented in Figure S2.

GPC was used to estimate the molecular weight of the prepared PLA composites. In order to validate the number average molecular weight (M_n_) values determined through GPC, the intrinsic viscosity [η] was also measured. The results are presented in Table 1. The presence of both organosolv lignin (oL) and organosolv lignin after ultrasonication ((US)oL) resulted in a decrease in the [η] values, as anticipated, since the hydroxyl groups of lignin functioned as initiation sites, resulting in the faster formation of inactive PLA chains [47]. Notably, two different M_n_ values were obtained in the presence of lignin. The smaller M_n_ value could be assigned to the rapid formation of linear PLA chains with a Ð > 2, whereas the larger M_n_ value could be attributed to the grafted PLA chains to lignin [25]. Grafting often leads to “star-like” polymers, with a lignin core and PLA chain arms [25,48]. These materials cannot be accurately measured by GPC due to their larger volume, but the high M_n_ is a strong indication that such structures were formed. Chile et al. proposed that in star polymers, M_n_ corresponds to the molecular weight of the grafted segment attached to a randomly chosen (lignin) core, while M_w_ reflects the average molecular weight of a randomly selected segment [49]. Importantly, unreacted lactide was not detected in any of the composite materials via NMR spectroscopy (Figure S3).

**Figure 2:**
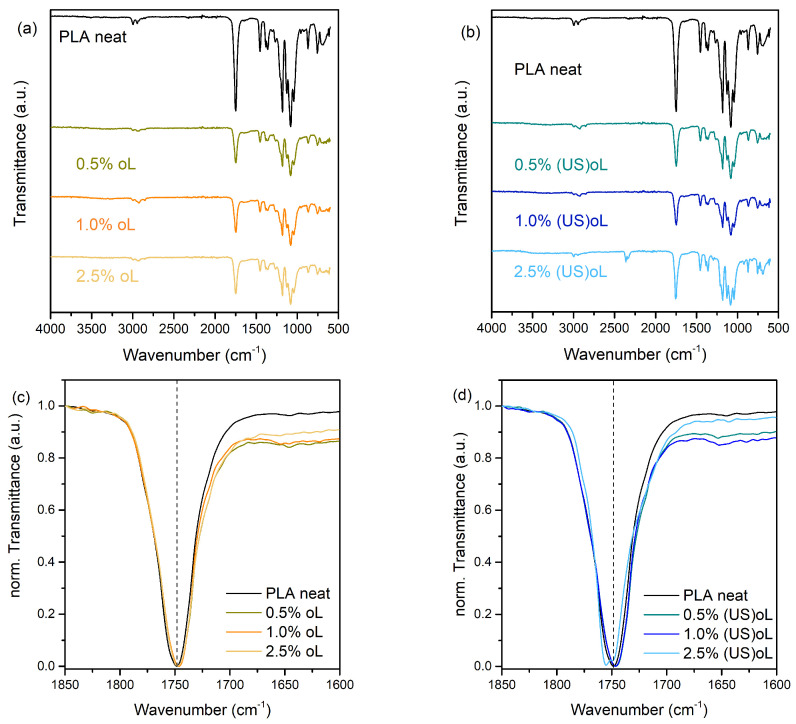
ATR-FTIR spectra of (**a**) PLA-oL and (**b**) PLA-(US)oL composites. Overlay of the corresponding ν(C=O) stretching bands after intensity normalization for (**c**) PLA-oL and (**d**) PLA-(US)oL composites.

The chemical structure and possible interactions in the composites were studied with FTIR spectroscopy. Figure 2a,b illustrates the ATR-FTIR spectra of neat PLA and the composite films with different lignin loadings. The FTIR spectrum of neat PLA presents a strong band at 1747 cm^−1^, characteristic of the ν(C=O) stretching vibration of the ester groups. The FTIR spectra of all the PLA composites with oL and (US)oL in the respective region of interest (1850 to 1600 cm^−1^) after intensity normalization are shown in Figure 2c,d. For all composites with pristine oL, a slight shift and a broadening toward lower wavenumbers was observed (Figure 2c). These trends were more evident for the composites prepared with (US)oL (Figure 2d), to the point where a shoulder was observed for the PLA–2.5% (US)oL composite. Such features in the FTIR spectra indicated an increasing fraction of PLA ester carbonyl groups interacting with the surface hydroxyl groups of lignin particles, either via hydrogen or covalent bonding, particularly in the case where (US)oL was introduced in the in situ ROP process [33,50,51,52,53,54].

To further investigate the interactions between the lignins and PLA, XPS spectra of neat PLA and PLA/lignin composites with a 2.5 wt% lignin content were recorded. The surface-wide scans of all samples showed the presence of C and O (Figure S4). Quantitative analysis revealed the atomic concentrations that are presented in Table 2.

For neat PLA, the C 1s and O 1s atomic concentrations were 69.9% and 26.5%, respectively, giving a C/O ratio of 2.64, as shown in Table 2. Upon the incorporation of 2.5 wt% oL, the C/O ratio decreased to 2.33, while for 2.5 wt% (US)oL it was slightly higher, at 2.38. These reductions in the C/O ratio could be directly related to the synthetic method as adding oxygen-rich lignin introduced functional groups such as hydroxyl, carbonyl, and ether groups into the composite. The ultrasonication treatment of lignin before its introduction in PLA did not alter its elemental composition significantly; however, it facilitated better dispersion and increased the availability of reactive hydroxyl groups.

**Figure 3:**
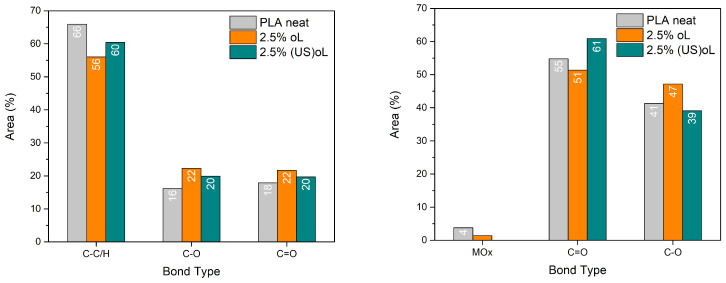
Percent area of the deconvoluted (**left**) C1s peaks and (**right**) O1s peaks of the XPS spectra.

High-resolution peak fitting was performed to resolve the chemical state of the elements. The deconvoluted C1s and O1s spectra are presented in Figure S5, and the results obtained by the fitting process (peak positions, % peak area, and bond type) are provided in Table S1. Figure 3 depicts the relative contribution of each C1s and O1s bond type.

After the incorporation of either of the lignins, the C1s % area of the C–C/H bonds decreased, while the area of the C–O and C=O bonds increased. Notably, the deconvolution of the O1s spectra revealed that the number of C=O bonds significantly increased when (US)oL was used. These changes suggested that more lignin–PLA ester bonds were formed when (US)oL was used, in agreement with the FTIR spectra. The reduced particle size of (US)oL led to an augmented specific surface area in comparison with oL, thereby increasing the availability of hydroxyl groups that served as grafting sites.

### 3.2. Lignin Dispersion in the Composites

**Figure 4:**
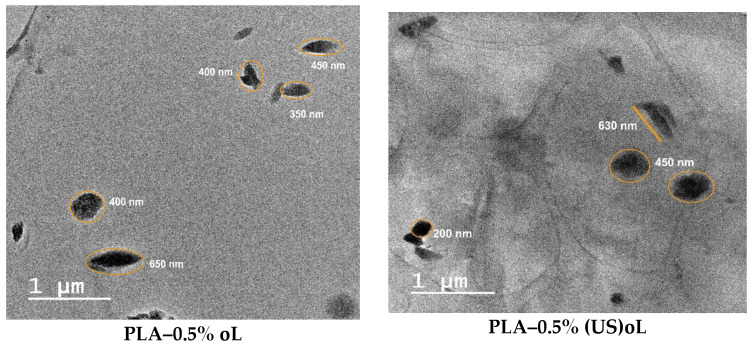

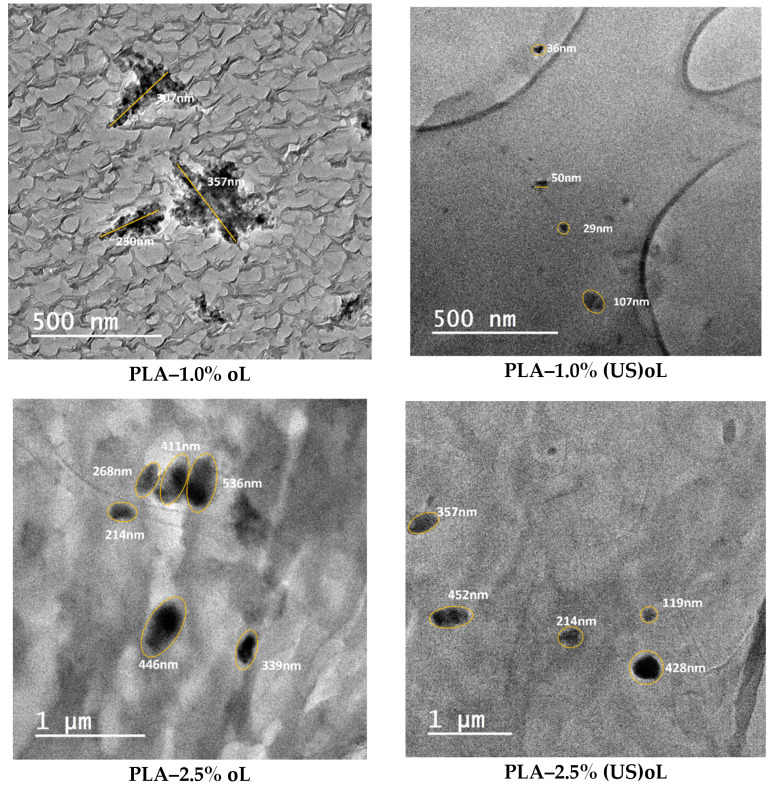
TEM micrographs of PLA–oL (**left**) and PLA–(US)oL composite films (**right**).

The dispersion of oL or (US)oL particles in the composites was assessed by TEM and SEM. The TEM images are shown in Figure 4, whereas the SEM images are provided in Figure S6. Both types of lignin were uniformly dispersed in PLA during the ROP of lactide. This verified the successful integration of the fillers into the PLA matrix and the interaction of lignins with the monomer, as expected by the in situ ROP process [25,34,55]. For all lignin contents, no large aggregates were observed, and the lignin particle size remained below 1 μm. However, the PLA–(US)oL composites consistently exhibited smaller lignin particle sizes, ranging from 30 to 450 nm, compared with the PLA–oL composites in which the lignin particle sizes ranged from 200 to 650 nm. Moreover, some clusters of individual particles were observed in the images of the PLA–oL composites. The finer dispersion of (US)oL nanoparticles in the PLA matrix was a direct consequence of their smaller particle size, as shown by DLS measurements (Figure S1). A similar trend was observed in the SEM images (Figure S6), where the PLA–oL composites showed larger particles dispersed throughout the PLA matrix compared with the PLA–(US)oL composites.

### 3.3. Mechanical Properties

**Figure 5:**
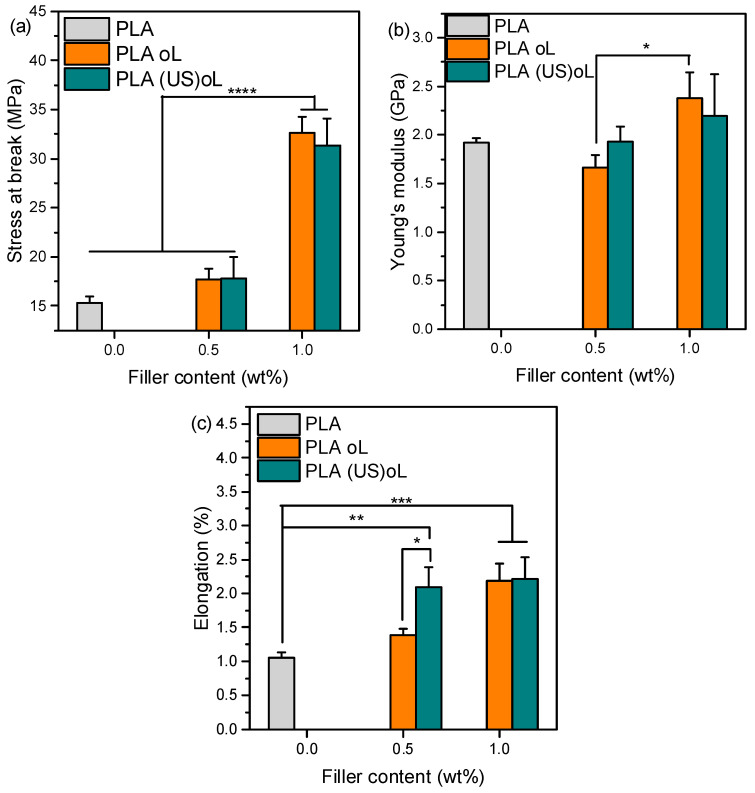
(**a**) Tensile stress at break, (**b**) elongation at break, and (**c**) Young’s modulus of neat PLA, PLA–oL, and PLA–(US)oL composites with 0.5 and 1 wt% organosolv lignin. One-way ANOVA with Tukey multi-comparison, * *p* ≤0.05, ** *p* ≤0.01, *** *p* ≤ 0.001, **** *p* ≤ 0.0001.

The mechanical properties of neat PLA and PLA-based composites were evaluated and compared using tensile testing. Figure 5 depicts the mechanical performance of neat PLA and the composites. The PLA–2.5% oL/(US)oL samples were not included in the results, because it was not possible to prepare a macroscopically homogeneous sample, suitable for tensile testing. A previous study reported that low lignin contents (~1 wt%) in PLA-g-lignin copolymers can enhance the tensile strength and elongation at break of PLA, whereas higher loadings (~4.5 wt%) tend to deteriorate these properties [38,45].

PLA exhibited tensile strength, elongation at break, and Young’s modulus of 15.29 MPa, 1.05%, and 1917 MPa, respectively (Figure 5). Adding 0.5 wt% of either of the lignins did not significantly affect the tensile strength or the Young’s modulus (*p* > 0.05). In contrast, the stress at break and elongation of the PLA–lignin composites with 1 wt% oL or (US)oL was significantly improved. In fact, the tensile strength was doubled, reaching approximately 36 and 32 MPa in the presence of 1 wt% oL and (US)oL, respectively (*p* ≤ 0.0001). This may be attributed to the better dispersion in polymeric matrix through ROP processing, as opposed to melt compounding [32].

The Young’s modulus of neat PLA (Figure 5b) did not show significant changes with the addition of either type of lignin of the fillers (*p* > 0.05). Only the increase in the content of oL from 0.5 to 1 wt% caused a significant increase (*p* ≤ 0.05).

The effect of lignins on the elongation at break of PLA-lignin composites (Figure 5c) was more pronounced; the addition of either of the lignins resulted in higher elongation at break compared with neat PLA. Interestingly, the addition of only 0.5 wt% of (US)oL significantly increased (*p* ≤ 0.05) the elongation at break in comparison with 0.5 wt% of oL. The positive impact of fillers on the elongation at break could be attributed to the presence of the grafted PLA chains that acted as compatibilizers [56]. Overall, (US)oL filler had a stronger impact on the elongation at break in comparison with oL, implying that the size reduction in lignin was beneficial when the goal was to increase the elongation of PLA. Importantly, for the composites containing 1 wt% lignin, elongation at break was doubled compared with neat PLA (*p* ≤ 0.001). The effect on the mechanical properties was less pronounced when microscale particles were used, indicating that size reduction and grafting acted synergistically to improve the elongation of aliphatic polyesters [38].

Previous studies reported an increase in elongation at break of PLA–lignin blends prepared by solvent casting and containing 5 wt% and 10 wt% PLA-grafted lignin NPs, but in both cases the tensile strength was reduced [27]. In this work however, the tensile strength was significantly increased.

### 3.4. Thermal Transitions and Crystallinity

**Figure 6:**
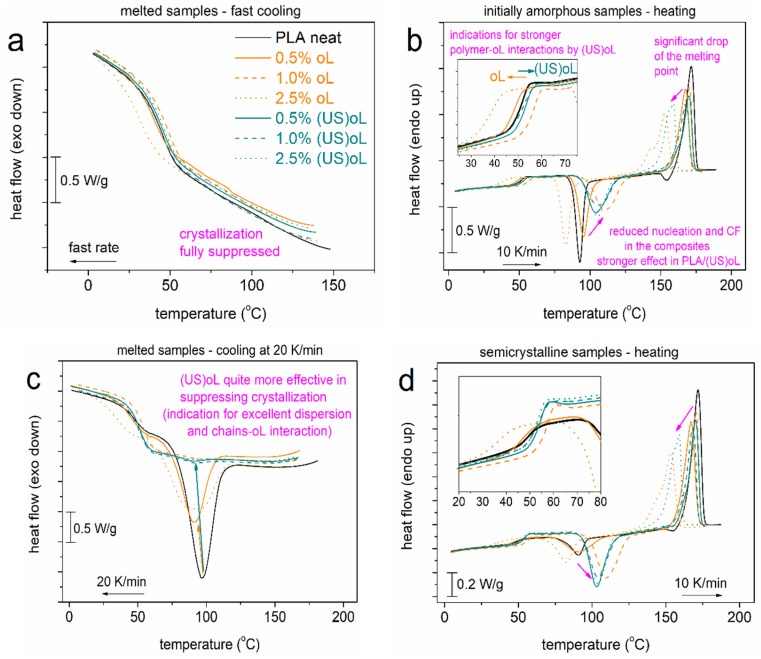
Comparative DSC traces for (**a**,**b**) scan 1 involving a fast cooling and heating at 10 K/min and (**c**,**d**) scan 2 involving a mild cooling at 20 K/min and heating at 10 K/min. The heat flow is shown upon normalization to the sample mass. The insets in (**b**,**d**) show the regions of glass transition in more detail, while the added arrows show effects imposed by the presence of oL and (US)oL.

In Figure 6, we present the compiled DSC data from the two scans performed. The most interesting values of these scans are listed in Table 2 and Table 3. During the fast cooling (Figure 6a), with the estimated rate being ~100 K/min, the crystallization was fully suppressed in all samples. During the subsequent heating (Figure 6b), all samples exhibited a glass transition (T_g_) step in a wide range (25–60 °C), a strong cold crystallization exothermal peak (75–130 °C), and a melting endotherm (130–180 °C). The systems exhibited a homogeneous character as no additional thermal event was recorded in the composites. The direct filler effect on the glass transition is shown in the inset to Figure 6b, wherein most PLA-oL systems exhibited a reduction in the T_g_, when compared with neat PLA. On the other hand, the PLA-(US)oL composites exhibited, interestingly, a slightly elevated T_g_. It has been shown in such systems that two major parameters contribute simultaneously to the T_g_: first, the lowering of M_n_, which tends to lower the T_g_, and second, the presence of interactions that tend to elevate the T_g_ [32] (and references therein). Consequently,, it may be deduced that the PLA–(US)oL interactions were the dominant parameter over that of M_n_. This was not true in the case of PLA–oL. The result was in accordance with the findings by FTIR, discussed in Section 3.1, where it was shown that the size reduction in the lignin led to an increased number of available -OH groups that initiated the ROP of lactide, resulting in more covalent bonds between PLA and lignin.

During the slower cooling in Figure 6c, the samples exhibited melt crystallization endothermal peaks, with the characteristic temperature position, *T*_c_, decreasing systematically in the PLA-oL/(US)oL composites, from 97 °C (neat PLA) down to 87 °C (PLA + 2.5% (US)oL). The same happened for the corresponding crystallization enthalpy, equivalently, for the crystalline fraction, CF_c_. Previously, in Figure 6b, we recorded a retardance of cold crystallization (elevation of T_cc_) and drop in the corresponding CF_cc_ (Table 2). In both cases of crystallization, the results indicated that the presence of oL and (US)oL, the corresponding interactions as well as the shortening of the polymer chains collectively resulted in a loss of crystallization nuclei (chain entanglements) and the reduced ability of polymer chains to form crystals (chain folding formation) [57,58]. The results were in agreement with previous findings on polymer nanocomposites exhibiting similar structural, dynamical and interaction changes [37,59,60].

Moving to melting, in Figure 6b,d and Table 3 and Table 4, the melting endotherm of PLA was quite homogeneous and located at T_m_ = 172 °C, whereas in the composites, T_m_ dropped, and the melting peak exhibited a more complex view (double structured). The effect reflected, in general, the drop in the crystals’ quality (density and size) related certainly to the drop of M_n_. Nevertheless, from the processing point of view, the T_m_ drop was wanted as it served well as a parameter that reduced the economic and environmental cost.

**Table 3:** Values of interest for DSC: glass transition temperature, T_g_; heat capacity change during glass transition, Δc_p_; cold crystallization temperature and enthalpy, Τ_cc_ and ΔH_cc_, respectively; and corresponding crystalline fraction, CF_cc_. Note: The CF_i_ values were estimated by comparing the corresponding ΔH_i_ values with the heat of fusion for PLA, taken as 93 J/g [61].

Sample	Melt Quenched (~90 K/min, Amorphous)				
	M_n_ (g/mol)	T_g_ (°C)	Δc_p_ (J/g∙K)	T_cc_ (°C)	CF_cc_ (wt)
Neat PLA	82k	51	0.51	93	0.42
PLA–0.5% oL	39k	48	0.52	95	0.41
PLA–1.0% oL	37k	56	0.50	111	0.45
PLA–2.5% oL	7k	38	0.53	83	0.42
PLA–0.5% (US)oL	52k	54	0.51	104	0.43
PLA–1.0% (US)oL	48k	52	0.53	107	0.40
PLA–2.5% (US)oL	24k	53	0.52	106	0.41

**Table 4:** Values of interest for DSC: glass transition temperature, T_g_; heat capacity change during glass transition, Δc_p_; cold crystallization temperature and enthalpy and melt crystallization temperature and enthalpy, Τ_c_ and ΔH_c_, respectively; corresponding crystalline fraction, CF_c_; and melting temperature and enthalpy, Τ_m_ and ΔH_m_, respectively. Note: The CF_i_ values were estimated by comparing the corresponding ΔH_i_ values with the heat of fusion for PLA, taken as 93 J/g [61].

Sample	Melt–Melt Crystallized (10 K/min, Semicrystalline)						
	M_n_ (g/mol)	T_c_ (°C)	CF_c_ (wt)	T_g_ (°C)	Δc_p_ (J/g∙K)	T_m_ (°C)	ΔH_m_ (J/g)
Neat PLA	82k	97	0.27	49	0.28	172	54
PLA–0.5% oL	39k	90	0.16	48	0.37	167	46
PLA–1.0% oL	37k	93	0.00	56	0.50	171	42
PLA–2.5% oL	7k	92	0.20	37	0.33	153	47
PLA–0.5% (US)oL	52k	90	0.01	53	0.50	170	41
PLA–1.0% (US)oL	48k	91	0.01	53	0.51	168	39
PLA–2.5% (US)oL	24k	87	0.01	53	0.53	159	42

**Figure 7:**
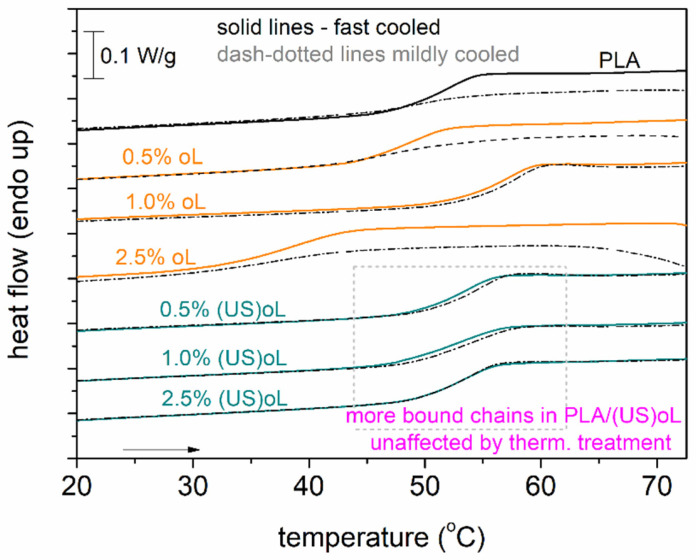
DSC heating (10 K/min) traces in the region of the glass transition for all samples, comparing the priorly fast and mildly cooled polymers. The shown heat flow has been normalized to the sample mass.

A final point regarding DSC concerns the actual mobility of the polymer chains and how it may be influenced by the presence of crystals. In Figure 7, we compare the T_g_ in the amorphous state (scan 1) with the semicrystalline state (scan 2). The marked area in Figure 7 shows that in PLA-(US)oL the glass transition step (position and height) was generally unaffected. The opposite observation was recorded for neat PLA and PLA-oL. The effect suggested that most probably, the polymer chains in PLA-(US)oL were less affected by the thermal/crystallization treatment or, in other words, were more bound or concentrated over the (US)oL entities than the oL ones. BDS is expected to shed more light on such issues as polymer mobility.

**Figure 8:**
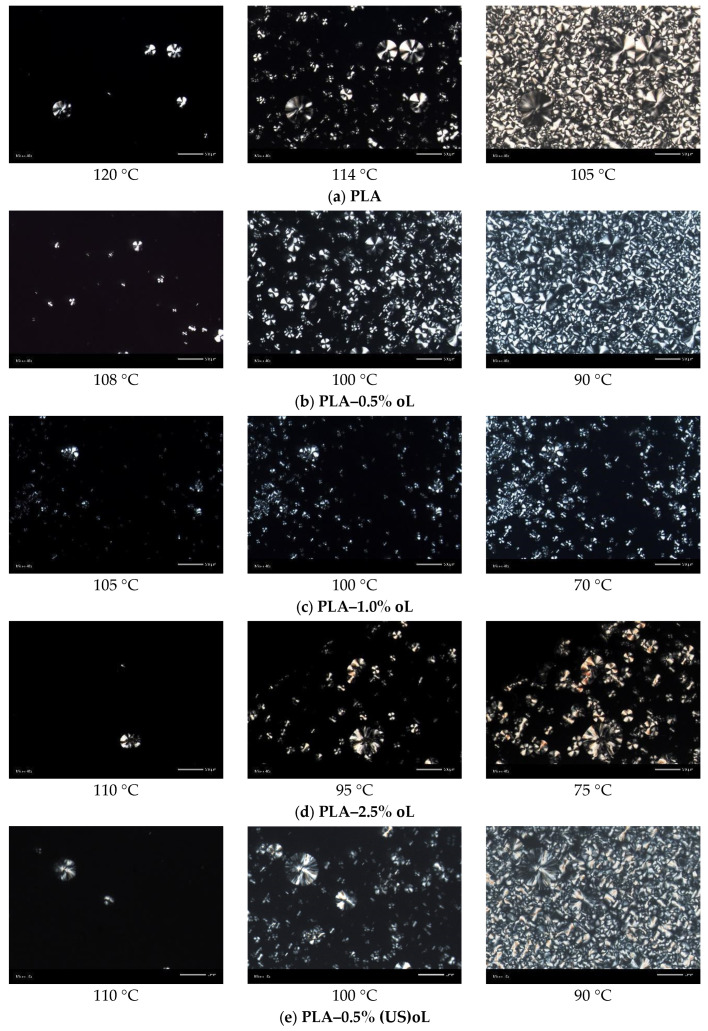

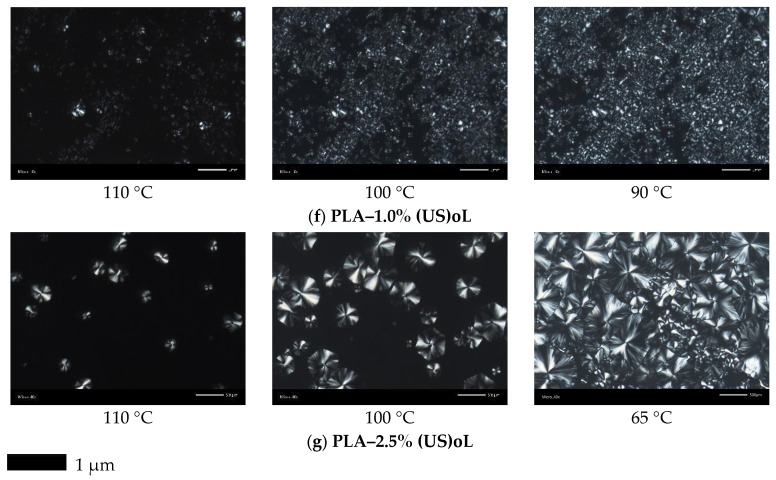
POM images during non-isothermal melt crystallization with a cooling rate of 10 °C/min. (**a**) PLA, (**b**) PLA–0.5% oL, (**c**) PLA–1.0% oL, (**d**) PLA–2.5% oL, (**e**) PLA–0.5% (US)oL, (**f**) PLA–1.0% (US)oL, and (**g**) PLA–2.5% (US)oL.

In Figure 8, the results by POM for samples melt-crystallized non-isothermally, in similar manner to scan 2 in DSC, are presented. From the initial crystallization stages (i.e., at the higher temperatures), the presence of lignin seemed to both promote at lower loadings, while hindering at higher loadings the nucleation. Direct effects of lignin type (treatment) on nucleation could not be easily extracted by POM, due to the large scale. The situation was clearer for the final crystallization state (right-side micrographs in Figure 8). For the low oL and (US)oL loadings, the semicrystalline morphology seemed similar between the composites and neat PLA, namely, it was quite dense consisting of a few large spherulites and many smaller ones. All the sample’s volume was filled by crystallites. On the other hand, when significantly increasing the lignin contents, the situation changed as the formed crystals were mainly small, with the exception of PLA-2.5% (US)oL. With the same exception, the semicrystalline morphology became looser in the PLA/lignin systems. This provided partial support to the findings of DSC on nucleation and crystal growth, as well as on the involvement of interfacial interactions (indirect effect).

From the points of view of processing and performance, the alternation in semicrystalline morphology can be exploited as a parameter for the tuning of various properties, e.g., the mechanical performance and the transport of small molecules (permeation) or, even, of heat [10,62]. In this sense, the addition of lignin and proper thermal treatment can be used as mediums for the indirect manipulations of properties. 

### 3.5. Broadband Dielectric Spectroscopy

**Figure 9:**
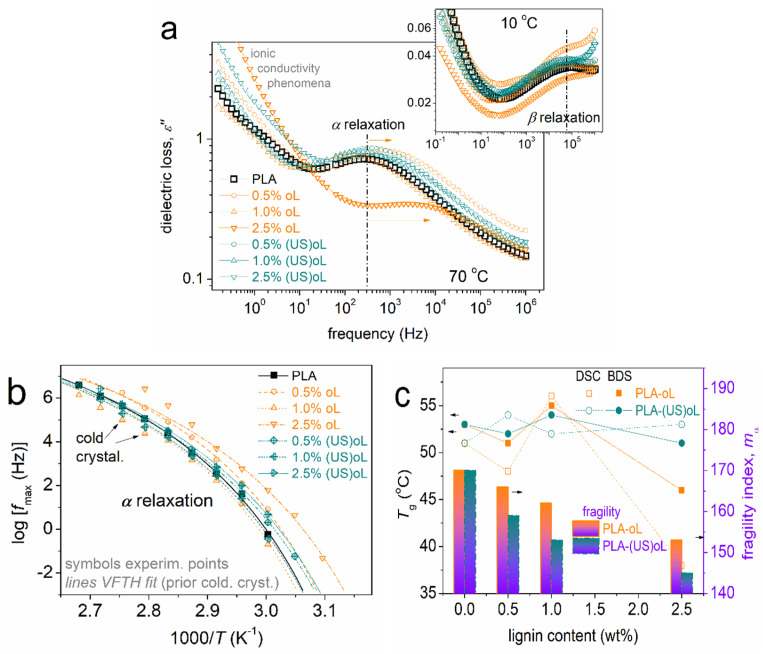
(**a**) Comparative isothermal ε″(f) curves at (main figure) 70 °C, i.e., above T_g_, and (inset) at 10 °C, i.e., below T_g_. The arrows mark the effects imposed on the position of segmental relaxation, related to the dynamics of glass transition. (**b**) The dielectric relaxation map for segmental dynamics. The added curved lines connecting the experimental points are fittings of the Vogel–Tammann–Fulcher–Hesse equation 53. (**c**) The lignin content dependence of the dielectric and calorimetric T_gs_.

Before proceeding with the results of BDS, we should provide a brief introduction on the recordings, physical aspects, and method of evaluation. In BDS, here, we present and evaluate the imaginary part of dielectric permittivity, ε″, which is associated with dielectric losses. ε″ changes significantly upon the activation of dipolar mechanisms, originating from actual molecular motions, either of local or of segmental character. These activated relaxation mechanisms are recorded as peaks in the ε″(f) spectra. Such are shown here in Figure 9a, at selected temperatures, providing a view of both the main segmental relaxation *α* and the local β relaxation (inset figure) related to the PLA ester bond (-C=O) motion [60,63]. The benefit of BDS is that the relaxation mechanism gradually moves toward higher frequencies within the f window as the temperature rises. This is because the relaxation times, τ_rel_, either decrease or the relaxation frequency maxima increase. It is essential that opposite to the “static” view of DSC, BDS enables the monitoring of the molecular “dynamics”.

As expected, the focus here is on the segmental α dynamics, namely, the dielectric analogue of the glass transition. We recall that the results correspond to initially amorphous samples (melted and fast cooled), in order to reveal any direct effects by the oL/(US)oL and the effects of M_n_.

In Figure 9a, a single segmental *α* relaxation is recorded in all samples, demonstrating mainly a mild acceleration in the composites as compared with the neat PLA matrix. As expected, the acceleration was accompanied by an increase in the ionic conductivity of transport throughout the matrix, an aspect that can be exploited within certain applications relevant to small-molecule transport (e.g., packaging).

The raw BDS data were critically analyzed by known routes [64,65] and widely used model functions, such as the Havriliak–Negami. Via this analysis we were able to construct the dielectric segmental relaxation map (Figure 9b) and further evaluate the time scale findings in terms of the dielectric T_g_ and the fragility index, m_α_ [66] (Figure 8c), namely, the measure of chain–chain cooperativity [66].

In the dielectric map of Figure 9b, a qualitative agreement with the calorimetric findings is revealed, i.e., the faster dynamics in the case of PLA-oL than that of PLA-(US)oL. Again, as discussed in the previous section, the effects suggested the domination of the strong interfacial PLA-(US)oL interactions as compared with those in PLA-oL. Contrary to DSC, the segmental dynamics in the composites were always faster than neat PLA. This has been observed before with other systems and can be explained in terms of dynamical heterogenies [67], contributing in different ways for the two in principle different techniques. Also, please note that BDS was able to capture the involvement of cold crystallization at the higher temperatures, recorded here as a disturbance in the time scale points (arrows).

One of the most interesting aspects revealed by BDS referred to the fragility index, m_α_ (Figure 9c). Fragility dropped in the composites, from ~170 in PLA down to ~145 in PLA + 2.5% (US)oL. The drop was monotonic with the lignin content, suggesting a decrease in the level of cooperativity. More interestingly, m_α_ was systematically lower in the PLA/(US)oL systems. The effect was compatible with the lowering of the chain length in the composites, resulting in a widening of the average cooperativity length, ξ, of the polymer chains not close to the lignins [61]. In addition, considering the overall findings, we could conclude that the polymer concentration was increased around the nanoparticles (denser around the smaller particles in PLA/(US)oL), and thus, the polymer density away from the nanoparticles decreased, or else the free volume increased. These results were expected to induce strong impacts on a series of materials performance, such as mechanical, heat transport, permeation of ions, and other small molecular (air, oxygen, and humidity).

### 3.6. Antioxidant Activity

**Figure 10:**
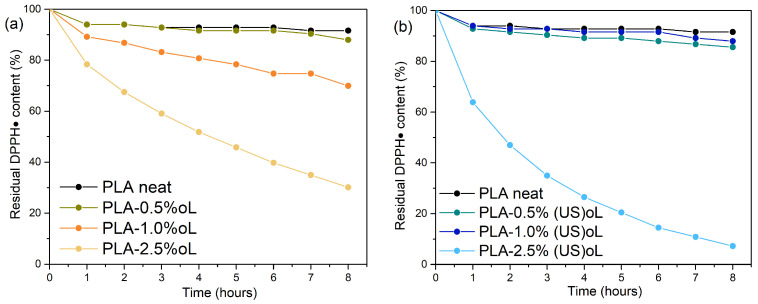
Reaction kinetics of the free radical DPPH during the immersion of (**a**) PLA–oL and (**b**) PLA–(US)oL composites in ethanol solutions.

The antioxidant capacity of lignin stems from the presence of phenolic hydroxyl groups in lignin’s structure, which serve as radical scavengers. These hydroxyl groups donate hydrogen atoms or electrons to neutralize free radicals, thereby preventing further oxidative reactions. In this study, the radical scavenging activity of composites was evaluated using the DPPH assay. The DPPH radical (DPPH•), a stable free radical with a characteristic purple color, reacts with phenolic groups to form a reduced, colorless state, which can be quantitatively monitored by UV-Vis [54,56,68]. Figure 10 presents the residual DPPH• content as a function of time for two sets of composites: PLA–oL (Figure 10a) and PLA–(US)oL (Figure 10b).

The neat PLA sample exhibited minimal antioxidant activity as it lacked phenolic groups responsible for scavenging radicals. The introduction of lignin into PLA enhanced the antioxidant properties of the composites in a dose-dependent manner. More specifically, the addition of 0.5 wt% oL did not affect the antioxidant capacity of PLA. However, when the oL content was increased to 1.0 wt% and 2.5 wt%, the respective PLA composites exhibited improved antioxidant activity, reaching residual DPPH• contents of 70% and 30% after 8h, respectively. Turning our attention to the PLA–(US)oL composites (Figure 10b), films containing 0.5 wt% and 1.0 wt% (US)oL displayed relatively weak antioxidant capacity, showing similar activity to that of neat PLA. However, the composites with a higher loading, specifically those containing 2.5 wt% of (US)oL, nearly eliminated DPPH•, leaving approximately 7% residual DPPH content after 8 h.

The mode of action of these composites as antioxidants was attributed to the presence of free phenolic hydroxyls, which interacted directly with radicals to interrupt oxidative chain reactions. Furthermore, when comparing these results with our previous work [25], where the composites were prepared by melt mixing, the antioxidant activity of the in situ ROP prepared composites was slightly improved. More specifically, for 2.5 wt% lignin content, the DPPH• residue was smaller herein than when using melt mixing. During ROP, it was mainly the aliphatic hydroxyls of lignin that initiated the reaction. Thus, the antioxidant activity was retained. The better dispersion and fewer aggregates of the lignin particles, facilitated by the ROP, improved the probability of free phenolic hydroxyls reacting with DPPH•. Thus, the in situ ROP approach allows of the retaining of the antioxidant properties of lignin in the composites.

### 3.7. Optical Properties

**Figure 11:**
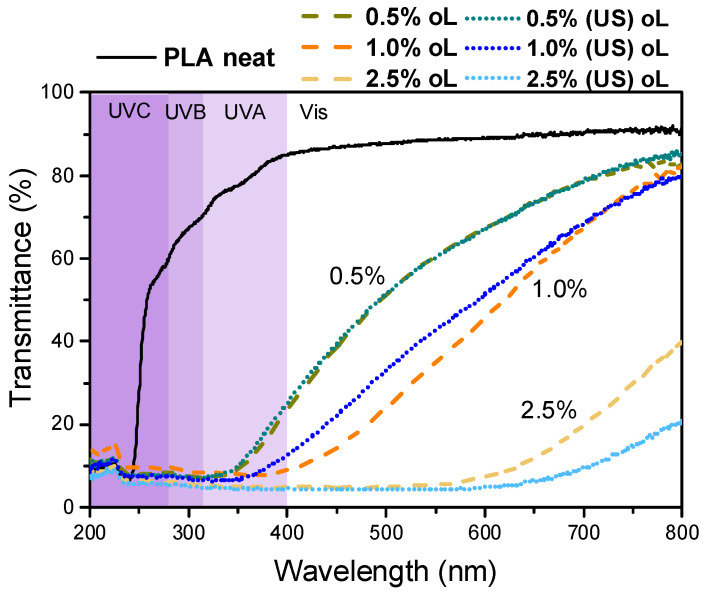
Optical properties of the neat PLA and PLA–oL/(US)oL composites.

The UV-Vis characterization of the produced materials was carried out, and the results for all composite films are presented in Figure 11. The PLA film was fully transparent in the UV-Vis region. However, upon the incorporation of either type of lignin, the transmittance in the visible region was reduced and was further decreased with increasing lignin content. The particle size of the lignin did not affect transparency in a measurable way.

The transmittance of all composites in the UV region and especially at wavelengths below 350 nm was negligible, highlighting the potential utility of PLA–lignin composites as fully biobased UV-blocking materials [40]. This phenomenon depended on the concentration of the filler as well. This significant reduction in UV transmittance could be attributed to the small particle size of the lignin particles and their even distribution due to the synthetic process. These factors contributed to the enhancement of UV-blocking properties of the composite films, as highlighted also in previous studies [40,41,56]. Overall, both PLA–0.5% oL and PLA–0.5% (US)oL retained adequate transparency while simultaneously blocking UV irradiation, an attractive feature for either food packaging or agricultural film applications.

## 4. Conclusions

In this study, PLA–organosolv lignin composites were successfully synthesized via in situ ring-opening polymerization (ROP) of L-lactide, using organosolv lignin as a filler at loadings ranging from 0.5 to 2.5 wt%. Ultrasonication was employed to reduce the lignin particle size, resulting in improved dispersion within the PLA matrix, as confirmed by SEM and TEM analyses. Strong interfacial interactions, particularly in the PLA–(US)oL composites, led to enhanced mechanical properties, including a two-fold increase in tensile strength and elongation at a 1 wt% loading. Antioxidant activity was significantly enhanced, with the PLA–2.5% (US)oL composite reducing residual DPPH by 93% after 8 h. Additionally, the UV-blocking capability of the composites, driven by lignin’s chromophoric properties, makes them promising candidates for sustainable applications. This work demonstrates the advantages of in situ ROP over conventional methods in achieving superior dispersion and functional properties. These findings highlight the potential of PLA–lignin composites for applications that require enhanced mechanical strength, antioxidant performance, and UV protection, such as in packaging and agricultural films.

## Figures and Tables

**Table 1:** Intrinsic viscosity and molecular weight values of PLA/oL and PLA/(US)oL composites via ROP.

Sample	[η] (dL/g)	M_n_ (g/mol)	Dispersity (Ð)
Neat PLA	1.53	79,000	2.8
PLA–0.5% oL	1.33	31,000	3.9
PLA–1.0% oL	1.11	19,400	2.2
		250,000	1.4
PLA–2.5% oL	0.39	10,800	2.5
		97,400	1.3
PLA–0.5% (US)oL	1.33	24,400	3.5
		500,000	1.6
PLA–1.0% (US)oL	1.20	28,123	2.0
		294,758	1.3
PLA–2.5% (US)oL	0.55	9100	1.9
		102,000	1.4

**Table 2:** Atomic concentrations of PLA and PLA–2.5% oL and (US)oL composites measured by XPS.

	C 1s	O 1s	
Sample	Atomic Ratio (%)	Atomic Ratio (%)	C/O Atomic Ratio
Neat PLA	69.9	26.5	2.64
PLA–2.5% oL	68.2	29.3	2.33
PLA–2.5% (US)oL	68.2	28.6	2.38

## Data Availability

The data of this study are included in the manuscript and Supplementary Material.

## Supplementary Materials

The following supporting information can be downloaded at https://www.mdpi.com/article/10.3390/polym16243542/s1: Figure S1: Particle size distribution of organosolv lignin, as received organosolv lignin, oL, and after ultrasonication treatment, (US)oL, measured via DLS. The respective hydrodynamic diameters reveal a reduction from approximately 1 μm (with a polydispersity index, PDI, of 25%) to around 700 nm (PDI = 24%) after US treatment, corresponding to a reduction in the lignin particle size by over 30%. Figure S2: Probable coordination–insertion ROP of LA catalyzed by Sn(Oct)2 with lignin as the source of -OH groups. Figure S3: The (a) 1H NMR and (b) 13C NMR (right) spectra of neat PLA and oL and (US)oL composites. Figure S4: XPS surface-wide scan of PLA and its composites with oL and (US)oL. Figure S5: Deconvoluted XPS spectra of (a) C 1s PLA, (b) O 1s PLA, (c) C 1s PLA–2.5% oL, (d) O 1s PLA–2.5% oL, (e) C 1s PLA–2.5% (US)oL, and (f) O 1s PLA–2.5% (US)oL. Figure S6: SEM micrographs of cryo-fracture cross-sections of PLA and its composites with oL (left) and (US)oL (right). Magnification x2500. The shown scale bar corresponds to the length of 10 μm. Table S1: Results of the peak fitting of the XPS spectra.
