# Supplementary material for: Lignin Particle Size Affects the Properties of PLA Composites Prepared by In Situ Ring-Opening Polymerization
Source: Polymers (Basel). 2024 Dec 19;16(24):3542. doi: 10.3390/polym16243542 (PMC11678871; doi:10.3390/polym16243542)
Supplement: Supplementary file 1 [file polymers-16-03542-s001.zip › polymers-3362607-supplementary.pdf]

Supporting information for:

Lignin Particle Size Affects the Properties of PLA Composites Prepared by In Situ Ring-Opening Polymerization

Sofia P. Makri [1,2], Eleftheria Xanthopoulou [2], Panagiotis A. Klonos [2,3], Alexios Grigoropoulos [1], Apostolos Kyritsis [3], Ioanna Deligkiozi [1], Alexandros Zoikis-Karathanasis [1], Nikolaos Nikolaidis [2], Dimitrios Bikiaris [2,*] and Zoi Terzopoulou [2,*]

[1] Creative Nano PC, 43 Tatoiou, Metamorfosi, 14451 Athens, Greece; s.makri@creativenano.gr (S.P.M.); a.grigoropoulos@creativenano.gr (A.G.); i.deligkiozi@creativenano.gr (I.D.); a.karathanasis@creativenano.gr (A.Z.-K.)

[2] Laboratory of Polymer and Colors Chemistry and Technology, Department of Chemistry, Aristotle University of Thessaloniki, 54124 Thessaloniki, Greece; exanthoa@chem.auth.gr (E.X.); pklonos@central.ntua.gr (P.A.K.); nfnikola@chem.auth.gr (N.N.)

[3] Dielectrics Research Group, Department of Physics, National Technical University of Athens, Zografou Campus, 15780 Athens, Greece; akyrits@central.ntua.gr

* Correspondence: dbic@chem.auth.gr (D.B.); terzozoi@chem.auth.gr (Z.T.)

Experimental

Scanning Electron Microscopy (SEM)

The morphology of cryofractured cross sections of the samples was studied using a JEOL JMS 7610 F (Freising, Germany) scanning microscope equipped with an energy dispersive X-ray (EDX) Oxford ISIS 300 micro-analytical system. A 200 Å thick carbon coating was applied to increase the conductivity of the samples.

Results

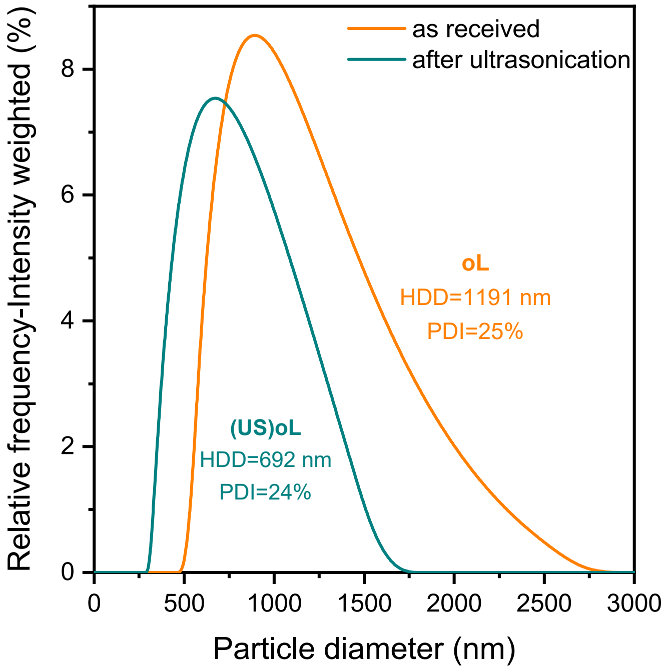

Figure S1. Particle size distribution of organosolv lignin, as received organosolv lignin, oL and after ultrasonication treatment, (US)oL measured via DLS. The respective hydrodynamic diameters reveal a reduction from approximately 1 µm (with a polydispersity index, PDI, of 25%) to around 700 nm (PDI = 24%) after US treatment, corresponding to a reduction of lignin particle size by over 30%.

Coordination-insertion:

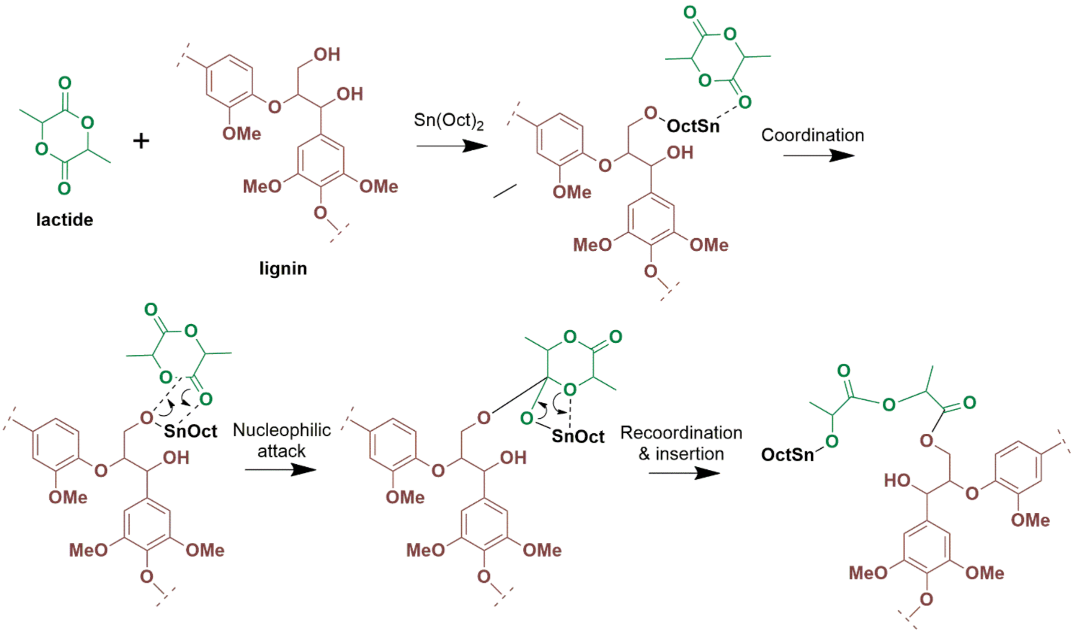

Propagation:

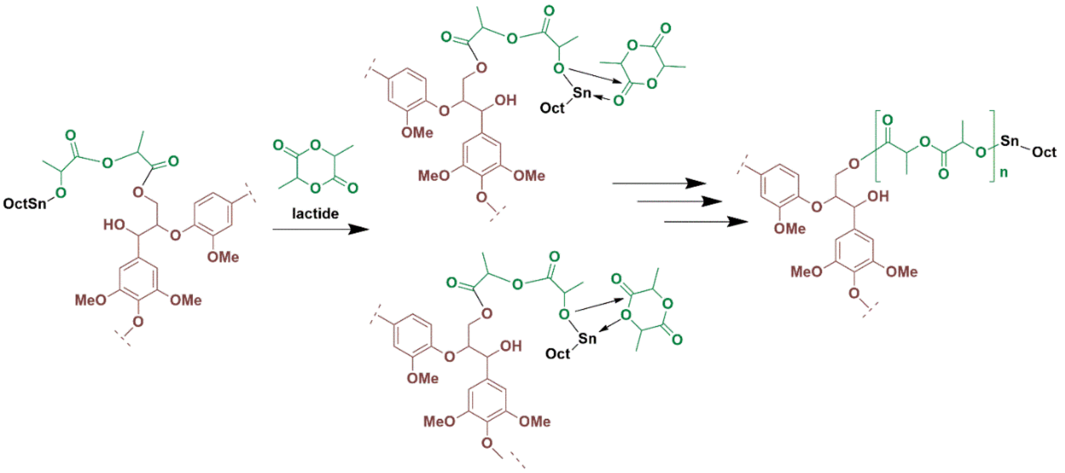

Termination:

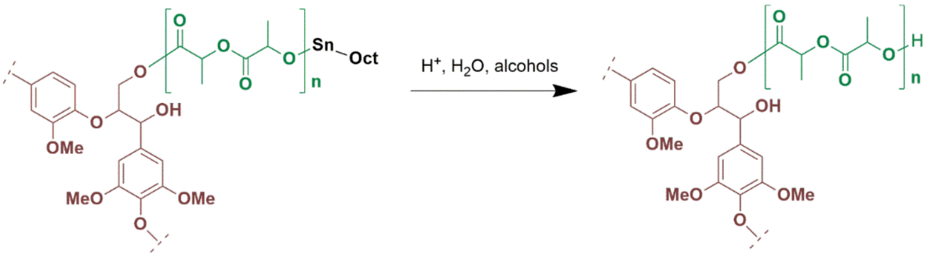

Figure S2. Probable coordination-insertion ROP of LA catalysed by $Sn(Oct)_2$ with lignin as the source of -OH groups.

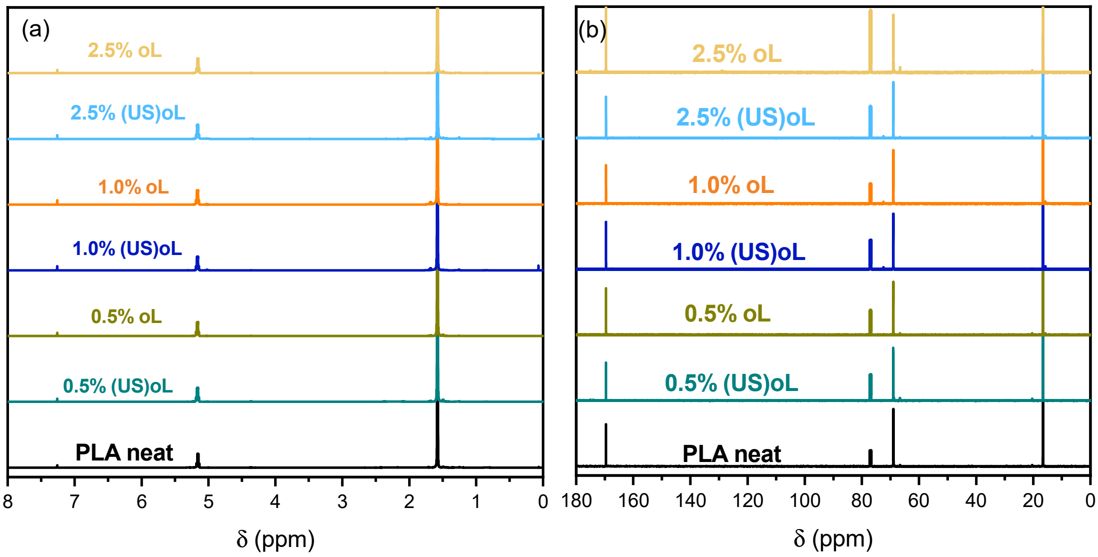

Figure S3. (a) ^{1}H NMR and (b) ^{13}C NMR (right) spectra of neat PLA and oL and (US)oL composites.

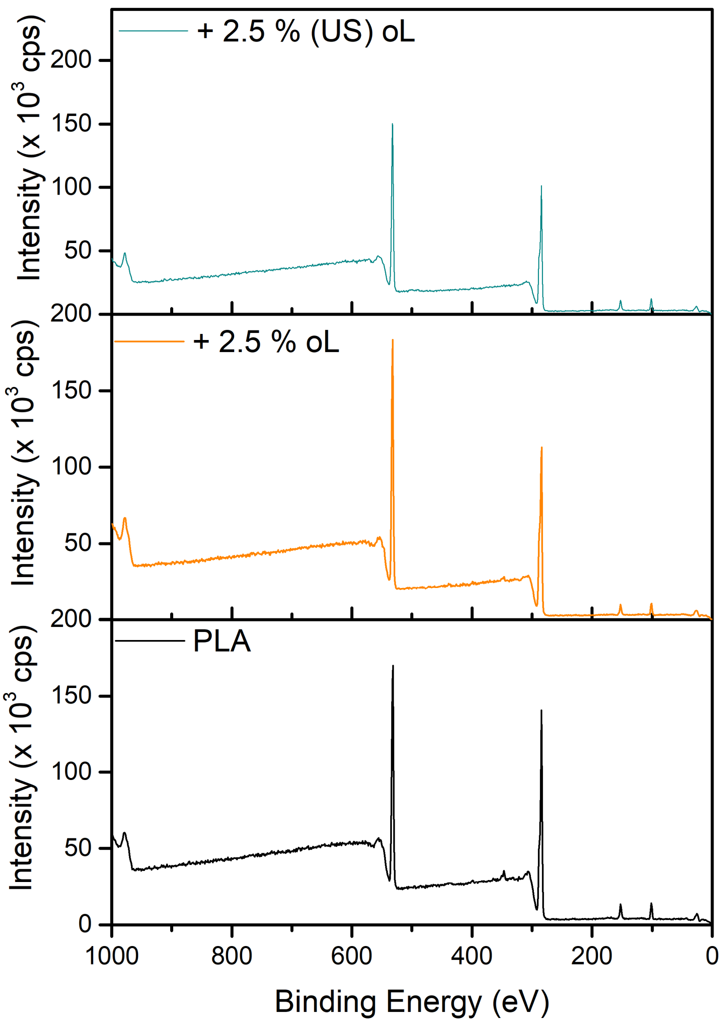

Figure S2. XPS surface-wide scan of PLA and its composites with oL and (US)oL.

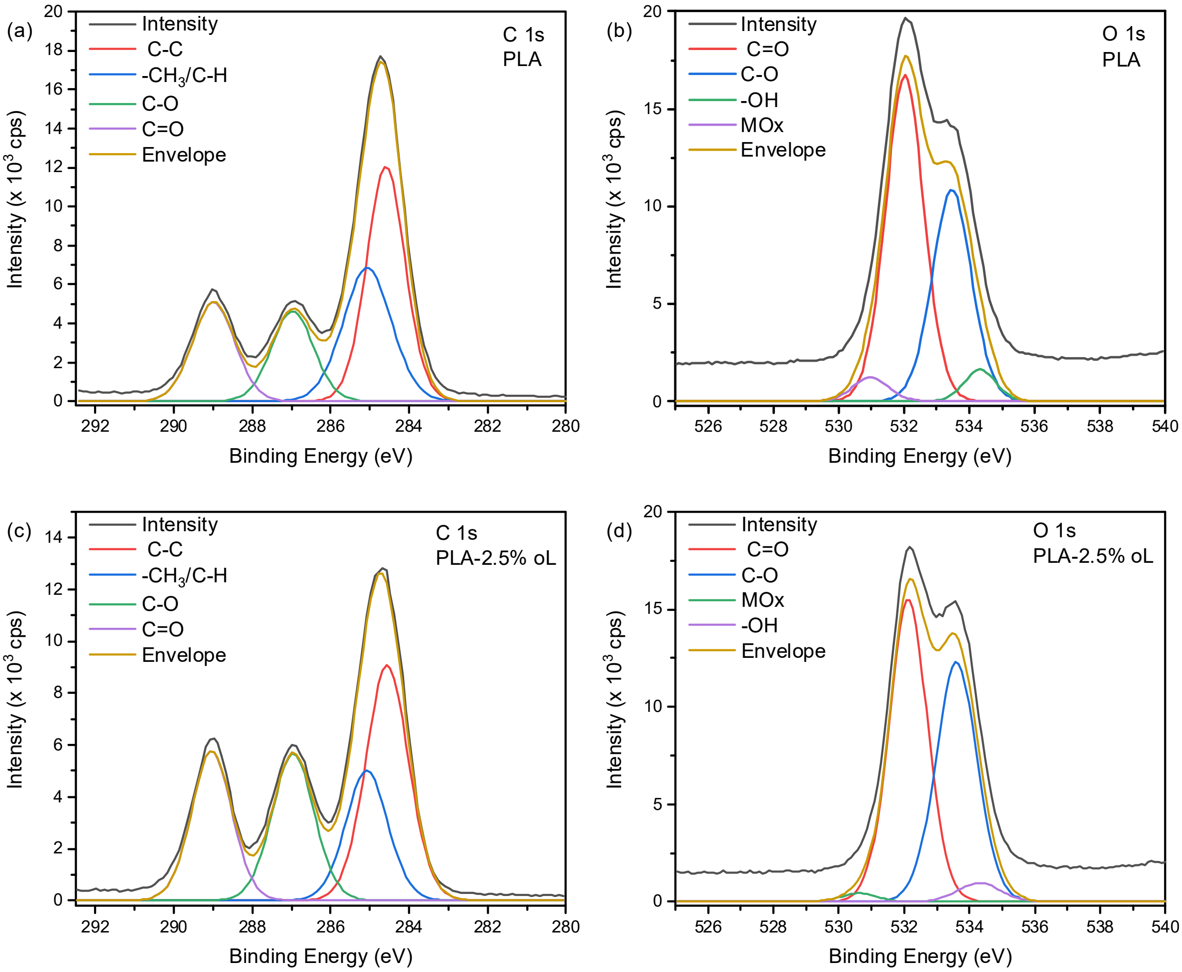

Figure S3. Deconvoluted XPS spectra of (a) C 1s PLA, (b) O 1s PLA, (c) C 1s PLA-2.5% oL, (d) O 1s PLA-2.5% oL, (e) C 1s PLA-2.5% (US)oL, (f) O 1s PLA-2.5% (US)oL.

Table S1. Results of the peak fitting of the XPS spectra.

Sample	C 1s			O 1s		
	Bond	Peak position (eV)	Area (%)	Bond	Peak position (eV)	Area (%)
PLA neat	C-C	284.6	38.4	C=O	532	54.8
	C-Hx	285.1	27.5	C-O	533.5	36.9
	C-O	286.9	16.2	-OH	534.3	4.4
	C=O	289.0	17.9			
PLA-2.5% oL	C-C	284.6	36.2	C=O	532.2	51.4
	C-Hx	285.1	19.8	C-O	533.6	43.6
	C-O	286.9	22.3	MOx	530.6	1.7
	C=O	289.0	21.7	-OH	534.4	3.6
PLA-2.5% (US)oL	C-C	284.6	40.4	C=O	532.2	60.9
	C-Hx	285.2	20	C-O	533.6	33.7
	C-O	286.9	19.9	-OH	534.3	5.4
	C=O	289.0	19.7			

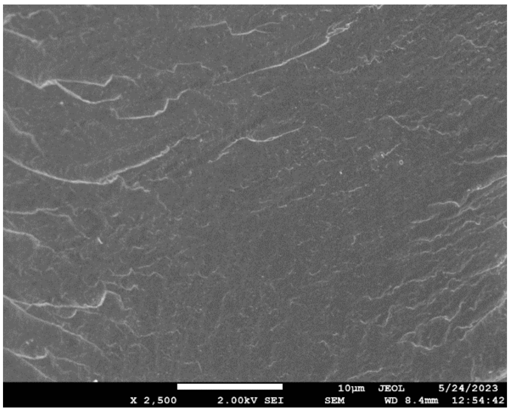

PLA neat

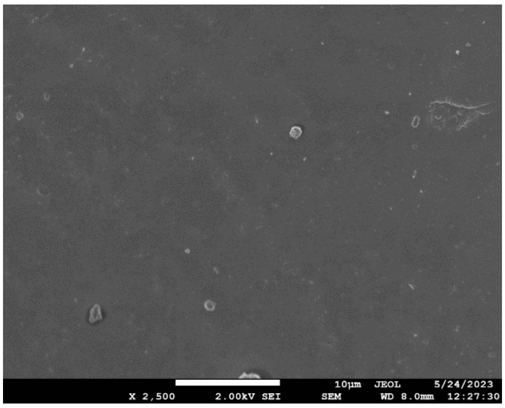

PLA-0.5% oL

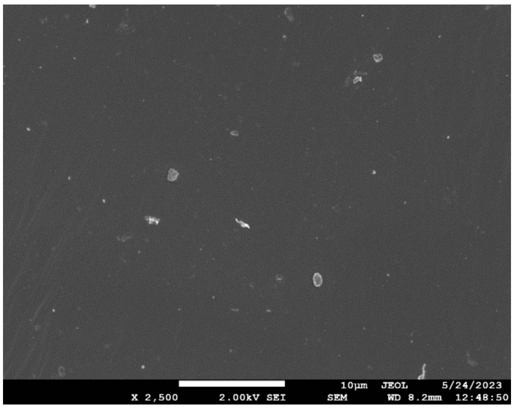

PLA-0.5% (US)oL

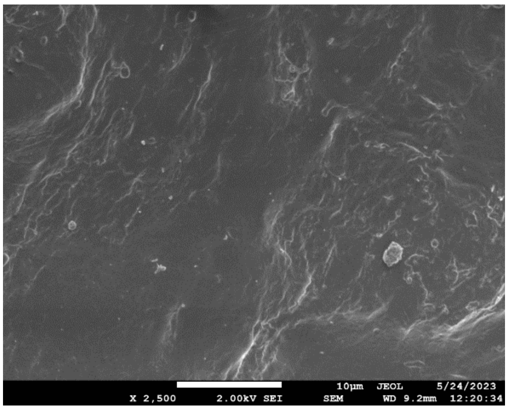

PLA-1.0% oL

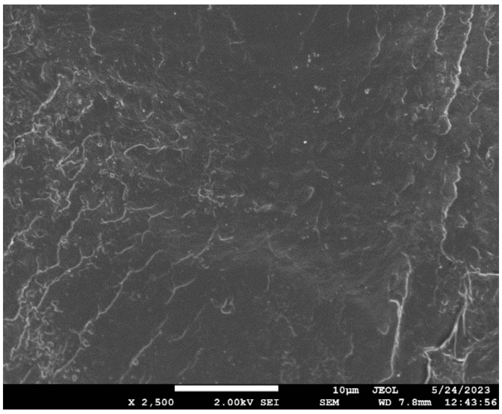

PLA-1.0% (US)oL

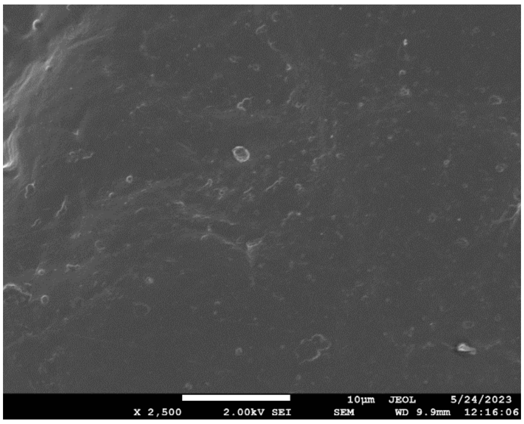

PLA-2.5% oL

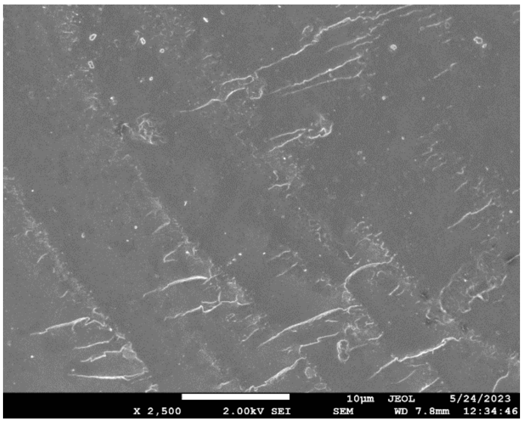

PLA-2.5% (US)oL

Figure S4. SEM micrographs of cryo-fractures cross-sections of PLA and its composites with oL (left) and (US)oL (right). Magnification x2,500. The shown scale bar corresponds to the length of 10 µm.
